# Identification of Variants Responsible for Monogenic Forms of Diabetes in Brazil

**DOI:** 10.3389/fendo.2022.827325

**Published:** 2022-05-03

**Authors:** Gabriella de Medeiros Abreu, Roberta Magalhães Tarantino, Ana Carolina Proença da Fonseca, Juliana Rosa Ferreira de Oliveira Andrade, Ritiele Bastos de Souza, Camila de Almeida Pereira Dias Soares, Amanda Cambraia, Pedro Hernan Cabello, Melanie Rodacki, Lenita Zajdenverg, Verônica Marques Zembrzuski, Mário Campos Junior

**Affiliations:** ^1^ Laboratory of Human Genetics, Oswaldo Cruz Institute, Oswaldo Cruz Foundation, Rio de Janeiro, Brazil; ^2^ Diabetes and Nutrology Section, Internal Medicine Department, Federal University of Rio de Janeiro, Rio de Janeiro, Brazil; ^3^ Laboratory of Immunopharmacology, Oswaldo Cruz Institute, Oswaldo Cruz Foundation, Rio de Janeiro, Brazil; ^4^ Laboratory of Genetics, School of Health Science, University of Grande Rio, Rio de Janeiro, Brazil

**Keywords:** monogenic diabetes, MODY, mitochondrial disease, genetic diagnosis, rare disorders, variants

## Abstract

Monogenic forms of diabetes mellitus may affect a significant number of patients of this disease, and it is an important molecular cause to be investigated. However, studies of the genetic causes of monogenic diabetes, especially in populations with mixed ethnic backgrounds, such as the one in Brazil, are scarce. The aim of this study was to screen several genes associated with monogenic diabetes in fifty-seven Brazilian patients with recurrence of the disease in their families and thirty-four relatives. Inclusion criteria were: Age of onset ≤ 40 years old, BMI < 30 kg/m², at least two affected generations and negative anti-GAD and anti-IA2 antibodies. MODY genes *HNF4A*, *GCK*, *HNF1A*, *HNF1B*, *NEUROD1*, *KLF11*, *PAX4*, *INS*, *KCNJ11*, and *MT-TL1* were sequenced by Sanger sequencing. We identified a total of 20 patients with variants, 13 GCK-MODY, four HNF1A-MODY, and one variant in each of the following genes, *HNF4A*, *HNF1B* and *MT-TL1*. Segregation analysis was performed in 13 families. Four variants were novel, two in *GCK* (p.(Met115Val) [c.343A>G] and p.(Asp365GlufsTer95) [c.1094_1095insGCGA]) and two in *HNF1A* (p.(Tyr163Ter) [c.489C>G] and p.(Val380CysfsTer39) [c.1136_1137insC]). Here we highlight the importance of screening for monogenic diabetes in admixed populations.

## Introduction

Diabetes is a clinically and genetically variable group of metabolic diseases characterized by hyperglycemia. It is estimated that monogenic forms of DM represent approximately 2% of cases with early onset diabetes (1, 2). These patients are frequently undiagnosed or misclassified as having type 1 DM (T1DM) or type 2 DM (T2DM). Monogenic diabetes includes different types of the disease: Neonatal diabetes mellitus (NDM) is a rare form of diabetes, characterized by the onset before six months of life (3). Mitochondrial diabetes, caused by a single alteration in the *MT-TL1* gene in mtDNA, in most cases by the m.3243A>G (4). And the most common form of monogenic diabetes, which is the Maturity-Onset Diabetes of the Young (MODY).

MODY is classically defined as a mild diabetes with autosomal dominant inheritance, early age at onset and impaired insulin secretion. In some cases, there is no insulin dependence (5). Since its first clinical description, variants in fourteen genes (*HNF4A*, *GCK*, *HNF1A*, *PDX1*, *HNF1B*, *NEUROD1*, *KLF11*, *CEL*, *PAX4*, *INS*, *BLK*, *ABCC8*, *KCNJ11* and *APPL1*) were described associated to this condition, presenting phenotypic, metabolic and genetic heterogeneity [review in (6)]. However, variants in *BLK*, *KLF11* and *PAX4* genes have been recently been challenged as causes of MODY (7). The frequency of variants in each gene is variable, according to the genetic background of the population and the methodology applied. In this study, we aimed to screen for variants in nine important genes associated to MODY, and the mitochondrial *MT-TL1*, in a sample with clinical characteristics of monogenic diabetes from Rio de Janeiro, Brazil to understand the contribution of each one of these genes in this cohort.

## Materials and Methods

### Patients

In this cross-sectional observational study were included patients who were treated at the Clementino Fraga Filho University Hospital and at the State Institute for Diabetes and Endocrinology Luiz Capriglione, from Rio de Janeiro, Brazil and their relatives. The inclusion criteria were patients with age at diagnosis (AAD) equal or less than 40 years old; positive family history of DM in at least two other generations in the family, or two or more first degree relatives at the same side of the family; and negative anti-GAD (Glutamic Acid Decarboxylase) and anti-IA2 (Islet Antigen 2) antibodies. The exclusion criteria were patients with T1DM with positive antibodies, obesity (Body Mass Index [BMI] ≥ 30 kg/m² or ≥ 95th percentile at AAD), history of diabetic ketoacidosis at diabetes onset, clinical signs of insulin resistance and presence of secondary causes the disease. Patients were divided into two groups, *GCK* or *HNF1A*, according to their clinical manifestation, since these two genes represents together the major cause of MODY. The GCK group (tested for the *GCK* gene) comprised patients most often asymptomatic that presented mild fasting hyperglycemia since birth ranging from 100 to 154 mg/dL, increase in glycaemia < 54 mg/dL after 75 g anhydrous dextrose and HbA1c < 7.5% (58 mmol/mol); and a evolutionarily stable disease (even without antidiabetic drugs); the remaining patients were included in HNF1A group (tested for the *HNF1A* gene).

Medical records were reviewed and participants were interviewed in order to obtain the following clinical information: AAD, duration of the disease, familiar history of DM, current and previous treatment, anthropometric measurements (height, weight and BMI), blood pressure, laboratory blood tests (Fasting Plasma Glucose [FPG], HbA1c, anti-GAD, anti-IA2, C-reactive protein [CRP], thyroid-stimulating hormone [TSH], free thyroxine 4 [Ft4], thyroid anti-peroxidase [TPO]), and presence of retinopathy, nephropathy, neuropathy and renal cysts. This study protocol was approved by The Ethics and Research Committee of the Clementino Fraga Filho University Hospital (CAAE n° 04232512.4.0000.5257) and by the State Institute for Diabetes and Endocrinology Luiz Capriglione (CAAE n° 04232512.4.3001.5266). All participants were informed about the aim of this study and provided a written informed consent.

**Figure 1:**
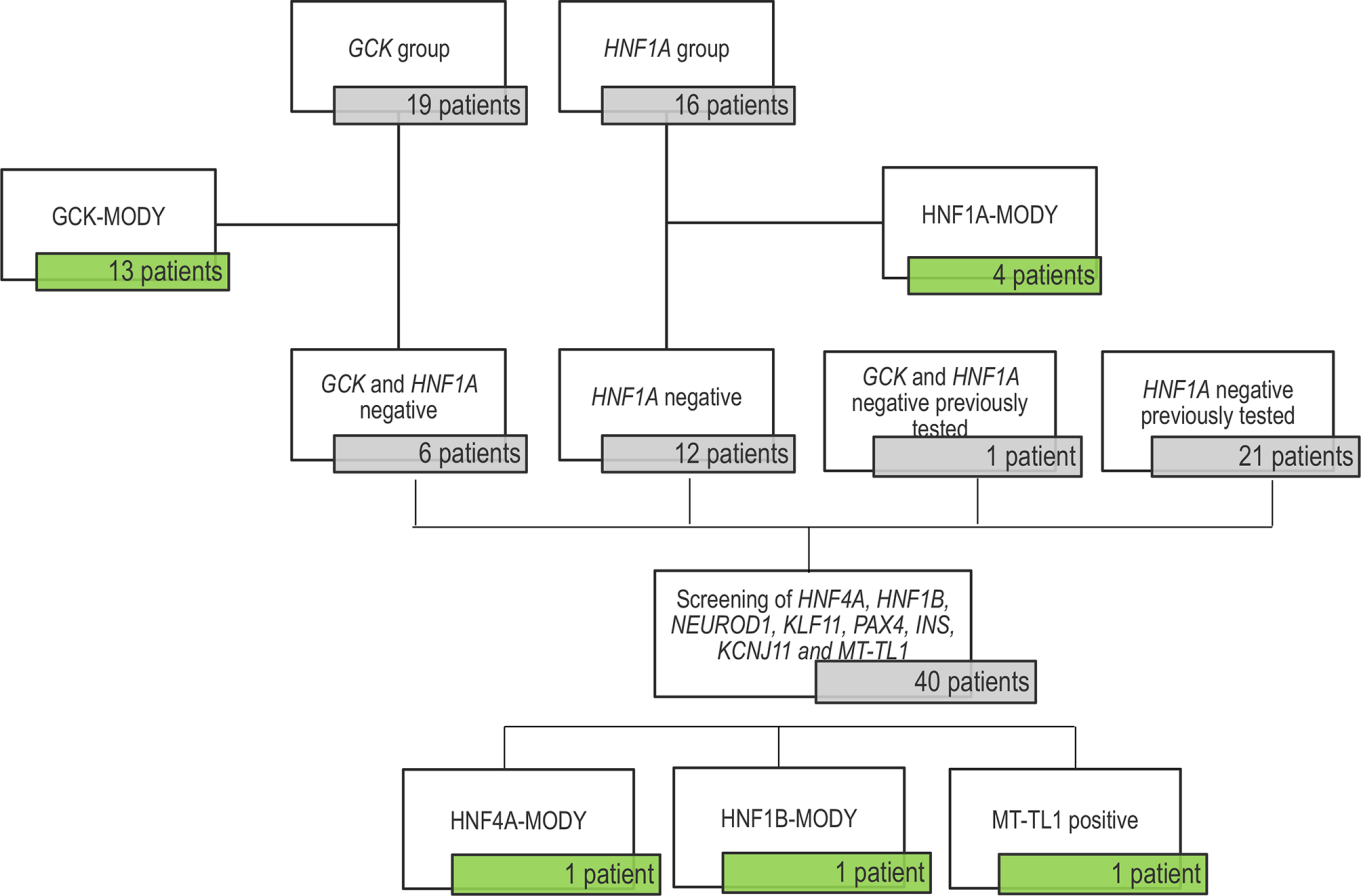
Flow diagram of the sample tested in this study. The untested patients included 19 patients in the GCK-MODY group and 16 patients for being tested for *HNF1A* variants. We found 13 *GCK* carriers and 4 patients with HNF1A-MODY. The 6 GCK negatives probands were screened for HNF1A variants and they were all negatives. This study also included a cohort previously search for variants in in the *HNF1A* gene (21 patients) with negative results; and one patient negative for both genes. Forty patients presented no variant in the two major genes (GCK and HNF1A) and were tested for *HNF4A*, *HNF1B*, *NEUROD1*, *KLF11*, *PAX4*, *INS*, *KCNJ11* and *MT-TL1*, resulting in the identification of three patients with variants.

Nineteen probands were enrolled in *GCK* screening and 16 were studied for *HNF1A*. In addition, twenty-one negative patients for *HNF1A* and one patient negative for *GCK*, previous reported by our group (8), were included for the screening of the other MODY genes. All negative patients for *GCK* variants were analyzed for *HNF1A* gene. After *GCK* and *HNF1A* sequencing, 40 patients with no detected variants were screened for mutations in *HNF4A*, *HNF1B*, *NEUROD1*, *KLF11*, *PAX4*, *INS*, *KCNJ11* and *MT-TL1*. A total of 34 relatives from 13 families were recruited 40 - 2.5%) (8 men and 16 women; average age 36.3 ± 20.7 years, ranging from 0 to 74 years), in which 21 presented DM and 13 did not report hyperglycemia. In total, 57 Brazilian probands were studied **(** **Table 1** **)**. The study sample formation is show in the **Figure 1**.

**Table 1:** Clinical description of the sample studied.

Variables	n	GCK-MODY group (n=20)	n	HNF1A-MODY group (n=37)
**Sex**	20		37	
women		10		22
men		10		15
**Age of diagnose (years)**	20	16.45 ± 11.53 (0,38)	37	18.16 ± 10.88 (2,37)
**Current BMI (kg/m²)**	19	21.54 ± 3.99	33	24.38 ± 3.44
**Fasting glucose (mg/dL)**	15	127.72 ± 23.08	17	150.64 ± 65.41
**Glycated hemoglobin (%)**	13	6.29 ± 0.66	31	7.73 ± 2.01
**Treatment**	20		37	
Insulin				
Yes		5		26
No		15		11
Oral hypoglycemic agents				
Yes		5		21
No		15		16
**Clinical diagnose suspicion**	18		35	
Without classification		2		6
Prediabetes		1		0
DM type 1		8		19
DM type 2		0		8
MODY		7		2

### Molecular Genetic Analysis

Genomic DNA from the probands was isolated from peripheral blood leukocytes using QIAamp DNA Blood Mini Kit (Qiagen, Hilden, Germany) and the genomic DNA from their relatives was collected and extracted from buccal epithelial cells according to the protocol in the literature (9). Primers were designed for all coding regions of 10 genes (*HNF4A*, *GCK*, *HNF1A*, *HNF1B*, *NEUROD1*, *KLF11*, *PAX4*, *INS*, *KCNJ11* and *MT-TL1*) using Primer3Plus software (http://www.bioinformatics.nl/cgi-bin/primer3plus/primer3plus.cgi). The information of the primers, PCR reagents and cycling conditions were summarized in **Supplementary Table S1**. PCR products were purified by ExoSAP-IT^®^ Reagent (Applied Biosystems, Vilnius, Lithuania), followed by Sanger sequencing reaction using the Big Dye Terminator Kit v3.1 (Applied Biosystems, Austin, TX, USA), conducted on an ABI 3130 Automatic Genetic Analyzer (Applied Biosystems). For variants considered to be likely pathogenic, orthogonal methodology was executed, including re-extraction of the sample, testing and sequencing of the forward and reverse strands of the area of interest a second time, and sequencing of the exon containing the variant identified in family members samples.

### Classification of Variants

Previous occurrence of all variants identified were checked in the follow public databases: PubMed, Clinvar, dbSNP (https://www.ncbi.nlm.nih.gov/), HGMD (http://www.hgmd.cf.ac.uk/ac/), gnomAD (https://gnomad.broadinstitute.org/) and the Online Archive of Brazilian Mutations (ABraOM; http://abraom.ib.usp.br/) (10). We classified the variants identified by our group according the published criteria of pathogenicity of the American College of Medical Genetics and Genomics and the Association for Molecular Pathology (ACMG/AMP) (Richards et al., 2015) available on VarSome (https://varsome.com/) (11).

Variant nomenclature followed the recommendations by the Human Genome Variation Society (http://www.hgvs.org/mutnomen/). Mitochondrial DNA nomenclature followed the Revised Cambridge reference sequence (rCRS) (http://www.mitomap.org/MITOMAP/HumanMitoSeq). Polymorphisms or synonymous variants reported are not included in this study. Variants not described in databases or published in scientific articles were referred as novel.

### Bioinformatics Analysis

The potential impact of the identified variants was tested by calculating prediction scores using eight prediction software: MutPred, FATHMM (v.2.3), VEST (v.4.0), SIFT, PolyPhen-2, Mutation Taster, PROVEAN, and Mutation Assessor. Conservation scores were calculated by five software: LRT, GERP++, SiPhy, PhastCons, and PhyloP. Revel was also used to scores of these thirteen software. All analysis were done by the Ensembl Variant Effect Predictor (VEP) and the results were retrieved from the dbNSFP.

More details of each software are present in **Supplemental Information**.

## Results

### Molecular Screening Findings

**Figure 2:**
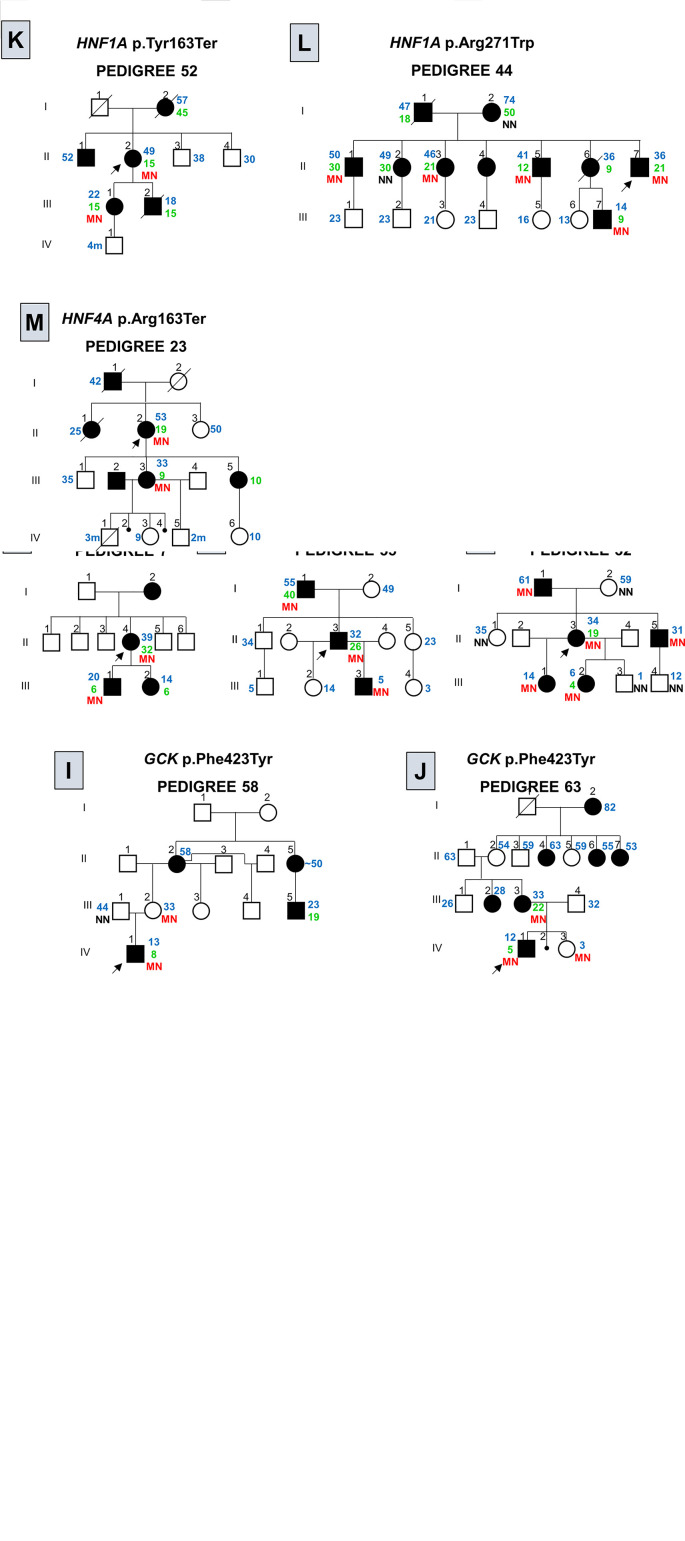
Pedigree of families with variants in genes associated to monogenic diabetes. Filled black symbols, grey symbols and empty symbols represent diabetic patients, impaired tolerance glucose patients and healthy individuals, respectively. Small black circles represent miscarriage [**(J)**, individual IV-2; **(M)**, individuals IV-2 and IV-4]. The present age of the individuals are show in blue and age at diagnosis in green, both are represent in years or months (m) when the age is followed by the **(M)**. Genotypes are expressed by homozygous normal allele (NN) and heterozygous mutated allele (MN) in red. Oblique lines through symbols represent deceased individuals. An arrow indicates the index case. In the pedigree 75 **(C)** < 25 indicates that the AAD was prior to the AAD usually observed for clinical criteria for MODY. In the pedigree 58 **(I)** the age of the individual II-5 was informed to be approximately (~) 50 years old. In the pedigree 23 **(M)** the family reported that the subject III-2 was diagnosed with MODY, although we are not able to confirm this information.

In the present study, we identified 20 patients with variants in our cohort: thirteen patients with variants in the *GCK* 40 - 2.5%) (3/19 - 68.4%), four patients with variants in the *HNF1A* (4/16 - 25%) and one variant in each of the following genes *HNF4A*, *HNF1B* and *MT-TL1* 40 - 2.5%) (/40 - 2.5%) (**Table 2**; **Supplementary Figure S1**). Clinical characteristics of the patients with variants described by our group are presented in **Supplemental Table 2**. We did not observe any variants in *KCNJ11*, *KLF11* and *INS* genes. Concerning the segregation study, 24 individuals from the 34 relatives showed the variant described in this study present in the probands. Among them, 20 individuals presented DM. Four relatives with variants in the *GCK* gene did not report DM at the moment of this study. Among the remaining ten relatives, one reported DM. Segregation analyses are shown in the **Figure 2**.

**Table 2:** Variants identified in the Brazilian sample described by our group.

Patient	Gene	GRCh38 location	Exon	RefSeq Gene position	Variant cDNA level	Variant protein level	Reference
P53	*GCK*	7:44153403	2	g.49768C>T	c.106C>T	p.(Arg36Trp)	(33)
P67	*GCK*	7:44153394	2	g.49777_49779del	c.115_117delAAG	p.(Lys39del)	(34)
P75	*GCK*	7:44153381	2	g.49790G>A	c.128G>A	p.(Arg43His)	(35)
P50	*GCK*	7:44153379	2	g.49792G>A	c.130G>A	p.(Gly44Ser)	(36)
P46	*GCK*	7:44152291	3	g.50880A>G	c.343A>G	p.(Met115Val)	Novel
P48	*GCK*	7:44149986	5	g.53185G>A	c.562G>A	p.(Ala188Thr)	(37)
P59	*GCK*	7:44149813	6	g.53358C>G	c.626C>G	p.(Thr209Arg)	(38)
P79	*GCK*	7:44149778	6	g.53393G>A	c.661G>A	p.(Glu221Lys)	(39)
P68	*GCK*	7:44145674	9	g.57497C>T	c.1076C>T	p.(Pro359Leu)	(40)
P55	*GCK*	7:44145666	9	g.57515_57518C>TinsGCGA	c.1094_1095insGCGA	p.(Asp365GlufsTer95)	Novel
P45, P58, P63	*GCK*	7:44145266	10	g.57905T>A	c.1268T>A	p.(Phe423Tyr)	(41)
P52	*HNF1A*	12:120988993	2	g.15248T>C	c.489C>G	p.(Tyr163Ter)	Novel
P44, P70	*HNF1A*	12:120994261	4	g.20516C>T	c.811C>T	p.(Arg271Trp)	(42)
P56	*HNF1A*	12:120996571	6	g.22826_22827insC	c.1136_1137insC	p.(Val380CysfsTer39)	Novel
P23	*HNF4A*	20:44413795	4	g.62995C>T	c.487C>T	p.(Arg163Ter)	(27)
P65	*HNF1B*	17:37731814	4	g.18293C>T	c.826C>T	p.(Arg276Ter)	(30)
P26	*MT-TL1*	m.3243A>G	-	-	-	-	(43)

Ensembl HGVS: GCK: ENSG00000106633.17, ENST00000403799.8, NM_000162.5, NP_000153.1, NG_008847.2; HNF1A: ENSG00000135100, ENST00000257555.11, NG_011731.2, NM_000545.8, NP_000536.5; HNF4A: ENSG00000101076, ENST00000316099.9, NG_009818.1, NM_000457.5, NP_000448.3; HNF1B: ENSG00000275410, ENST00000617811.5, NG_013019.2, NM_000458.4, NP_000449.1; MT-TL1: NC_012920, gi:251831106, AC_000021.2.

### GCK-MODY (OMIM # 125851)

*GCK* screening showed 11 different variants in 13 probands: seven men and six women. Two of the observed *GCK* variants were novel, the p.(Met115Val) (c.343A>G) and the p.(Asp365GlufsTer95) (c.1094_1095insGCGA); variant p.(Met115Val) was reported in gnomAD (Frequency: 0.00001193), however, no phenotype characteristics were available. The *missense* p.(Met115Val) variant was observed in a female patient (P46) (**Figure 2E**; individual III-3) diagnosed in her first pregnancy at the age of 25 years. This variant was observed segregating from her father (**Figure 2E**; individual II-2) with diabetes and was found in her two brothers (**Figure 2E**; individuals III-1 and III-2). The patient P55 (**Figure 2G**; individual II-3), harboring the *frameshift* p.(Asp365GlufsTer95) variant, was diagnosed at 26 years of age in a routine exam (BMI at diagnosis: 22.3 kg/m²). At the age of 32 years, when he entered in this study, he showed FPG varying from 109 to 128 mg/dL and HbA1c of 6.1% and he was controlling his hyperglycemia with diet. This variant was also observed in his father (**Figure 2G**; individual I-1) diagnosed at 40 years old and in his 5-year-old son (**Figure 2G**; individual III-3), diagnosed at birth. His son was born prematurely, before 32 weeks of gestation. Both, the proband and his son, control their glycemic level with nutritional diet.

### HNF1A-MODY (OMIM # 600496)

Among the 16 patients screened for *HNF1A*, four presented variants, one man and three women. The screening of *HNF1A* revealed two novel variants, one *frameshift*, p.(Val380CysfsTer39) (c.1136_1137insC), and one *nonsense*, p.(Tyr163Ter) (c.489C>G). They were absent in gnomAD and ABraOM population databases. The p.(Val380CysfsTer39) *frameshift* variant was found in a patient (P56) diagnosed at 35 years, with a BMI at diagnostic of 28.2 kg/m². At the time of this study, at the age of 36 years (BMI: 25.3 kg/m²), she showed a FPG of 133 mg/dL and HbA1c of 6.9%, (TSH 0.73 mIU/L; Ft4 of 1.4 ng/dL). She has been treated with alogliptin (25 mg/day). She reported eight family members with DM: her father and her mother (AAD: 30 and 50 years old, respectively), one brother (AAD: 40 years old), two sisters (AAD: 32 and 36 years old), two nieces and one nephew with AAD before the age of 25 years. The family members were not available to participate in our study. The novel *nonsense* p.(Tyr163Ter) variant was observed segregating from the patient P52 (**Figure 2K**; individual II-2) to her daughter (**Figure 2K**; individual III-1). The patient AAD was 15 years old. She presented polyuria, polydipsia and weight loss. Glyburide was initiated and she maintained good glycemic control for almost 26 years and at the age of 41 years, her treatment was switched for insulin (NPH and Regular) and Metformin XR (2000 g/day). At the time of this study, at the age of 49 years, she had a BMI of 25.8 kg/m², and HbA1c of 8.34%. She presented retinopathy and hypertension. The patient had continued the treatment with insulin (0.76 U/kg/day), and Metformin XR (2000 g/day) and gliclazide was started (60 mg/day). The patient’s daughter was diagnosed at 15 years old and has been treated with gliclazide (120 mg/day) and insulin (30 U/day).

### HNF4A-MODY (OMIM # 125850)

A *nonsense* variant in the exon 4 of *HNF4A*, p.(Arg163Ter) (c.487C>T) was found in a woman (P23), the AAD was 19 years in a routine exam (BMI at diagnosis: 20 kg/m²) (**Figure 2M**; individual II-2). At the age of 49 years, she presented a FPG of 294 mg/dL, managed with OAD (Glimepiride 4 mg/day) and, at the age of 53 years, she was initiated on insulin therapy. The variant segregated to her daughter (**Figure 2M**; individual III-3), with AAD of 9 years old, and FPG of 125 mg/dL. The proband’s daughter has 24 years of diagnosis and presents polyneuropathy, renal insufficiency and retinopathy, and has been treated with insulin therapy (10 U/day) with poor glycemic control. The proband’s daughter gave birth to a baby (**Figure 2M**; individual IV-1) with congenital heart malformation who died with three months of life. Later she also had two miscarriages (**Figure 2M**; individuals IV-2 and IV-4) and two children (**Figure 2** **M**; individuals IV-3 and IV-5). Her son (**Figure 2M**; individual IV-5) was born with birth weight of 1.400 g and breathing problems. The children and their fathers were not available for testing. The proband’s younger daughter (**Figure 2M**; individual III-5) also presents DM, diagnosed at the age of 10 years old. However, she was not available for test.

### HNF1B-MODY (OMIM # 137920)

The screening of the entire coding region of *HNF1B* showed a *nonsense* variant in the exon 4, p.(Arg276Ter) (c.826C>T). The patient (P65) is a woman of 19 years old, clinically diagnosed with T1DM at 14 years old with symptoms of decompensated diabetes (at diagnosis: FPG > 500 mg/dL; BMI 23.6 kg/m²). At the moment of this study, she presented a BMI of 21.9 kg/m², a HbA1c of 12.1%, and she was on insulin therapy (1.7 U/kg/day). The patient was initiated on insulin therapy since her diagnosis and presents no complications of DM; she reports nephrolithiasis. Furthermore, it was informed that her father, uncle and grandfather had DM, and both her uncle and grandfather deceased in young age from DM complications.

### *MT-TL1* (OMIM * 590050)

The variant m.3243A>G in the *MT-TL1* gene was identified in a male patient (P26) with clinical diagnosis of T1DM at 28 years old (BMI at diagnosis: 21.9 kg/m²). At entry in the study, he was 30 years old, showing an HbA1c of 5.6%, FPG of 116 mg/dL (2-hour postprandial glucose: 145 mg/dL), plasmatic fasting insulin of 5.1 mcU/mL (postprandial insulin: 17.7 mcU/mL) and C-peptide of 2.1 ng/mL. He reported that his mother, father and eight siblings had DM. He did not show any sensorineural hearing loss. At the age of 37 years, he has been treated with insulin (0.12 U/kg/day) and gliclazide (60 mg/day). His family was not available for testing.

### Bioinformatics Analysis

The 16 different variants identified in genomic DNA in this study were evaluated through *in silico* predictions algorithms. All ten *missense* variants were predicted as pathogenic at least for seven of the nine programs used to predict the pathogenicity. Besides, they were predicted as conserved at least for three out of four conservation predictions algorithms (**Supplemental Table S3**). According to the ACMG classification of pathogenicity, from the 16 variants in genomic DNA described by our group, ten were characterized as pathogenic and 6 variants as likely pathogenic (**Supplemental Table S4**).

## Discussion

In this study, patients were screened firstly for the *GCK* or *HNF1A* genes, according with their phenotypic manifestation, and then for *HNF4A*, *HNF1B*, *NEUROD1*, *KLF11*, *PAX4*, *INS*, *KCNJ11* and *MT-TL1*. We observed 13 cases of GCK-MODY and 4 cases of HNF1A-MODY, respectively. Concerning the others MODY subtypes, we found one variant in each *HNF4A*, *HNF1B*, and *MT-TL1* (**Table 1**).

Until now, variants in 14 genes have been recognized to cause monogenic diabetes type MODY, and variants on *GCK* and *HNF1A* represent the major cause worldwide. The frequency of each MODY subtype varies according to recruitment criteria and genetic background (6). In UK, variants in *HNF1A* were responsible for 52% of all MODY cases, followed by 32% of GCK-MODY (12). A retrospective database study of MODY cases reported in Brazil described GCK-MODY as the most common form, followed by HNF1A-MODY (13). Depending on the genetic cause, each MODY subtype may present a different clinical profile, with variable age at onset, treatment response, and with some subtypes having a higher risk of long-term complications of DM, and extra pancreatic manifestations (14).

In our GCK-MODY cohort, the age at diagnosis ranged from 9 months to 26 years (**Supplemental Table S1**) and the majority of the patients were diagnosed in routine exams. This findings was expected since the diagnosis is usually incidental and at any age (15). Among our 13 GCK-MODY patients, four were diagnosed in their childhood as having T1DM, three of them were treated with insulin therapy. Out of these 13, four patients were being treated with OAD at the moment of the study. Misdiagnosis of GCK-MODY patients or misclassification as T1DM are frequent (16), which reinforce the importance to screen patients with clinical phenotype for GCK-MODY. Therefore, after our molecular diagnostic analysis, six patients stopped allpharmacological treatment. Typically, patients with GCK-MODY with less than 40 years old show an HbA1c ranging from 5.6 to 7.3% and a FPG range of 5.6 to 8.0 mmol/L (17). Our patients had the HbA1c (6-7.2%) and FPG (6-7.7 mmol/L) within these ranges.

The screening of the coding region of *GCK* revealed 11 different variants; the likely pathogenic missense variants p.(Arg36Trp), p.(Ala188Thr), p.(Glu221Lys), and p.(Phe423Tyr) were previously described by other groups in Brazilian probands (13, 18–20). The p.(Phe423Tyr) was the only one to appear in more than one patient, it was found in three unrelated probands (P45, P58 and P63). To the best of our knowledge, this is the first report of the *GCK* p.(Arg43His), p.(Gly44Ser), p.(Thr209Arg), and p.(Pro359Leu) *missense* variants in Brazilian patients. We observed the first two segregating in their families (**Figures 2C, D**). Unfortunately, both P59 (p.(Thr209Arg)) and P68 (p.(Pro359Leu)) proband’s families were not available for analysis. Among the observed variants in the *GCK*, two were novel, the *missense* p.(Met115Val) (c.343A>G) and the *frameshift* p.(Asp365GlufsTer95) (c.1094_1095insGCGA). The p.(Met115Val) is registered in dbSNP under the number rs771677681 and is found in gnomAD with an allele frequency of 0.00001193; however, this variant was not reported associated to diabetes in the databases. This variant was observed in the proband P46 (**Figure 2E** individual III-3) and in her two older brothers, inherited from their father with diabetes; the proband’s middle brother did not report hyperglycemia, and his blood tests were not available. The p.(Asp365GlufsTer95) was observed segregating with DM through three generations (**Figure 2G**).

Variants in the *HNF1A* gene usually have high penetrance and patients are usually diagnosed between the ages of nine to 40 years (21, 22). The age at diagnosis of our patients ranged from 14 to 35 years old (**Table 1**). Patients with hyperglycemia caused by *HNF1A* variant can remain sulfonylurea responsive for many years (23). Patients P44 and P56 were switched from insulin to OAD, after 15 years and 1 year since their diagnosis, respectively. However, some patients require insulin treatment, as is the case for patient P52.

The screening of *HNF1A* showed three different variants;. The *HNF1A* p.(Arg271Trp) (c.811C>T), found in two unrelated patients (P44 and P70) segregates among several individuals with DM from the P44 proband’s family (**Figure 2L**). The P70 proband’s family was not available. This variant seems to decrease the HNF1A affinity and binding to DNA (24, 25). We also found a novel *nonsense* variant p.(Tyr163Ter) (c.489C>G), segregating from the patient P52 to her daughter with diabetes (**Figure 2K**), both diagnosed at the age of 15 years old. Additionally, we found a novel *frameshift* variant p.(Val380CysfsTer39) (c.1136_1137insC) in the patient P56; the P56 patient’ family was not available. According to ACMG guidelines the HNF1A p.(Tyr163Ter) and p.(Val380CysfsTer39) are classified as pathogenic and p.(Arg271Trp) as likely pathogenic, supporting the evidence of these variants as the cause of diabetes in these families.

Variants in the *HNF4A* gene seem to be a rare cause of monogenic diabetes in Brazil (26). The *nonsense* variant p.(Arg163Ter) was described by Lindner and coworkers (1997) as the second variant found in the *HNF4A* associated to MODY in a family from German ancestry (27). The patients from the reported family were treated by OAD or insulin, and patients with longer time of the disease showed retinopathy and peripheral polyneuropathy (27). This variant was found in our study in the proband P23 (**Figure 2M**; individual II-2), segregating to her daughter (**Figure 2M**; individual III-3). The HNF4A works as a heterodimer and the premature stop codon results in a truncated protein that loses the transactivation domain (27, 28) and the ability to form heterodimers (29). Laine and coworkers (2000) observed the same variant with a dominant-negative effect (28). Lindner and coworkers (1997) reported patients with the same variant with coronary heart disease and no nephropathy (27). The family members in our study reported renal insufficiency and no heart disease. The proband’s daughter (**Figure 2M**; individual III-3) presented peripheral polyneuropathy.

The *nonsense HNF1B* p.(Arg276Ter) was described in two Japanese patients with small kidneys and multiple renal cysts (30, 31). More information from this patients and renal ultrasound was not available, however a review of her medical chart revealed nephrolithiasis.

In this study, we also described one male patient carrying the *MT-TL1* m.3243A>G variant. The patient did not present any other clinical manifestations of MIDD, as sensorineural hearing loss. It has been described a wide spectrum of clinical variability for *MT-TL1* m.3243A>G variant, ranging from asymptomatic carriers to lethal multisystem disorders (32). These findings highlight the importance of screening this genetic variant in patients with familiar recurrence of diabetes despite the lack of other clinical characteristics related to MIDD and MELAS syndrome.

This study has some limitations worth noting: 1) small sample size; 2) DNA samples from relatives were extracted from saliva, different from the DNA source of the patients (blood) and it may cause false negatives due to somatic mosaicism; 3) segregation analysis of *HNF1A* p.(Val380CysfsTer39) and *GCK* p.(Met115Val) were not possible in unaffected relatives to ensure absence of variant and were also not possible in some of the families; 4) by using sanger sequencing instead of NGS we were unable to test other rare genes associated to monogenic diabetes commonly present in NGS panels and we cannot rule out the presence of large duplications and deletions; 5) not all rare forms of diabetes were included here, e.g. neonatal diabetes;

Here we highlight the importance of screening for monogenic forms of diabetes in patients with familial history of diabetes. An accurate diagnosis with molecular confirmation of monogenic diabetes can improve the choice for a better therapeutic management of patients and their families.

## Data Availability Statement

The datasets presented in this study can be found in online repositories. The names of the repository/repositories and accession number(s) can be found in the article/**Supplementary Material**.

## Ethics Statement

The studies involving human participants were reviewed and approved by The Ethics and Research Committee of the Clementino Fraga Filho University Hospital (CAAE n° 04232512.4.0000.5257) and by the State Institute for Diabetes and Endocrinology Luiz Capriglione (CAAE n° 04232512.4.3001.5266). Written informed consent to participate in this study was provided by the participants’ legal guardian/next of kin.

## Author Contributions

GA: Conceptualization, methodology, validation, formal analysis, investigation, writing- original draft, and project administration. RT: conceptualization, methodology, resources, writing - review and editing, supervision, and project administration. AF: Resources, writing - review and editing. JA: Methodology and investigation; RBS: Software, formal analysis, and writing - review and editing. CS: Investigation and writing - review and editing. AC: Writing - review and editing. PC: Resources, writing - review and editing, funding acquisition, and supervision. MR: Resources, writing - review and editing, funding acquisition, and supervision. LZ: Resources, writing - review and editing, funding acquisition. VZ: Resources, writing - review and editing, and funding acquisition. MCJ: Resources, writing - review and editing, supervision, funding acquisition, and project administration. All authors contributed to the article and approved the submitted version.

## Conflict of Interest

The authors declare that the research was conducted in the absence of any commercial or financial relationships that could be construed as a potential conflict of interest.

## Publisher’s Note

All claims expressed in this article are solely those of the authors and do not necessarily represent those of their affiliated organizations, or those of the publisher, the editors and the reviewers. Any product that may be evaluated in this article, or claim that may be made by its manufacturer, is not guaranteed or endorsed by the publisher.

## Supplementary Material

Supplementary Figure 1 Electropherograms of variants probably pathogenic found by Sanger sequencing among Brazilian patients with suspicious of monogenic diabetes. Novel variants are showed in blue. An arrow indicates the position of the variant in the electropherogram.
