# Supplementary figures and images for: Identification of Variants Responsible for Monogenic Forms of Diabetes in Brazil
Source: Front Endocrinol (Lausanne). 2022 May 3;13:827325. doi: 10.3389/fendo.2022.827325 (PMC9110842; doi:10.3389/fendo.2022.827325)

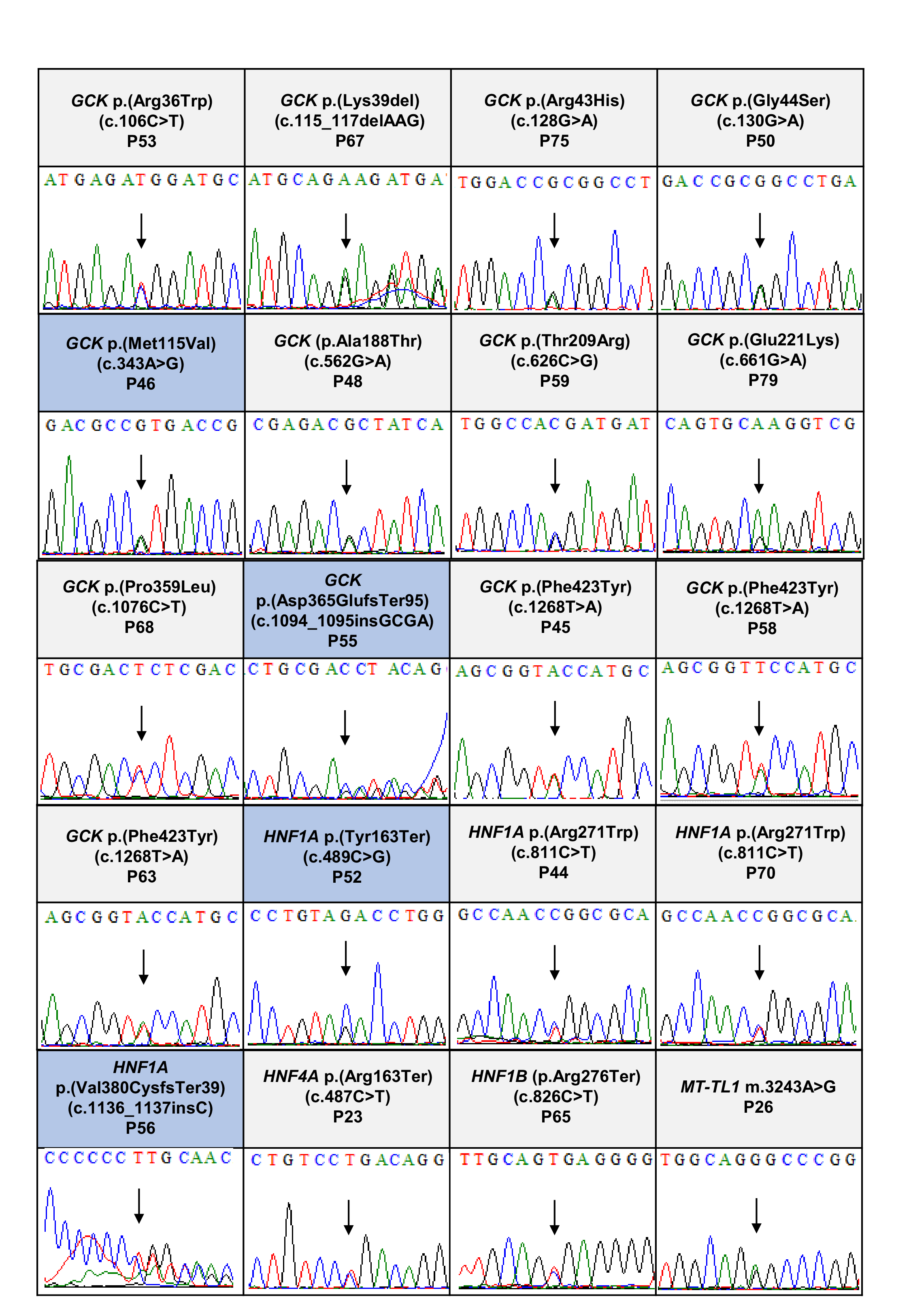

Supplement: Supplementary Figure 1 — Electropherograms of variants probably pathogenic found by Sanger sequencing among Brazilian patients with suspicious of monogenic diabetes. Novel variants are showed in blue. An arrow indicates the position of the variant in the electropherogram. [file Image_1.tiff]
