# Supplementary material for: Identification of Variants Responsible for Monogenic Forms of Diabetes in Brazil
Source: Front Endocrinol (Lausanne). 2022 May 3;13:827325. doi: 10.3389/fendo.2022.827325 (PMC9110842; doi:10.3389/fendo.2022.827325)
Supplement: Supplementary file 2 [file DataSheet_1.docx]

**Supplemental information**

**Bioinformatics analysis**

The potential impact of the identified variants in this study and variants described in our previous studies (1–4), was tested by calculating prediction scores: MutPred (http://mutpred.mutdb.org/) (5), FATHMM (v.2.3) (http://fathmm.biocompute.org.uk/) (6), VEST (v.4.0) (http://cravat.us/CRAVAT/) (7,8), SIFT (https://sift.bii.a-star.edu.sg/) (9), PolyPhen-2 (http://genetics.bwh.harvard.edu/pph/index.html) (10), Mutation Taster (http://www.mutationtaster.org/) (11), PROVEAN (http://provean.jcvi.org/index.php) (12), and Mutation Assessor (http://mutationassessor.org/r3/) (13). For Conservation scores were calculated by LRT (http://www.genetics.wustl.edu/jflab/lrt_query.html) (14), GERP++ (http://mendel.stanford.edu/SidowLab/downloads/gerp/) (15), SiPhy (http://portals.broadinstitute.org/genome_bio/siphy/) (16), PhastCons and PhyloP (http://compgen.cshl.edu/phast/) (17) were used. Revel (https://sites.google.com/site/revelgenomics/) was applied to scores of these thirteen software (18). All analysis were done by the Ensembl Variant Effect Predictor (VEP) (https://www.ensembl.org/Tools/VEP) (19) and the results were retrieved from the dbNSFP (database for nonsynonymous SNP’S functional predictions) (20,21).

To predict the effect of the splice variant in the acceptor site, eight *in silico* analyses were performed. Scores prediction for the mutant allele were obtained from Adaptive Boosting Algorithm (ADA) (22), Random Forest (RF) (23) and MaxEntScan (24) tools provide by VEP (https://www.ensembl.org/Tools/VEP) (29) and the results were retrieved from the SNV splicing consensus regions database (dbscSNV) (25). While, NetGene2 (26,27) and HMM-gene (v.1.1) (28) (https://services.healthtech.dtu.dk/), NNSplice (v.0.9) (https://www.fruitfly.org/seq_tools/splice.html) (29), FSPLICE (v.1.0) (http://www.softberry.com/berry.phtml?topic=fsplice&group=programs&subgroup=gfind), and Human Splicing Finder (v.3.1.) (HSF) (https://www.genomnis.com/access-hsf) (30) generate their prediction score for the wild-type allele and one for the mutant allele separately.

1. Tarantino RM, Abreu G de M, Fonseca ACP de, Kupfer R, Pereira M de FC, Campos M, et al. MODY probability calculator for GCK and HNF1A screening in a multiethnic background population. Arch Endocrinol Metab [Internet]. 2019 Sep 25;1(8):1–7. Available from: http://www.scielo.br/scielo.php?script=sci_arttext&pid=S2359-39972019005008101&lng=en&nrm=iso

2. de Medeiros Abreu G, de Almeida Pereira Dias Soares C, Magalhães Tarantino R, Proença da Fonseca AC, Bastos de Souza R, Carvalho Pereira M de F, et al. Identification of the First PAX4-MODY Family Reported in Brazil. Diabetes, Metab Syndr Obes Targets Ther [Internet]. 2020 Jul;Volume 13:2623–31. Available from: https://www.dovepress.com/identification-of-the-first-pax4-mody-family-reported-in-brazil-peer-reviewed-article-DMSO

3. Abreu G de M, Tarantino RM, Cabello PH, Zembrzuski VM, Fonseca ACP, Rodacki M, et al. The first case of NEUROD1‐MODY reported in Latin America. Mol Genet Genomic Med [Internet]. 2019 Dec 2;7(12):1–6. Available from: https://onlinelibrary.wiley.com/doi/abs/10.1002/mgg3.989

4. Abreu GDM, Tarantino RM, da Fonseca ACP, de Souza RB, Soares CAPD, Cabello PH, et al. PDX1-MODY: A rare missense mutation as a cause of monogenic diabetes. Eur J Med Genet [Internet]. 2021 May;64(5):104194. Available from: https://linkinghub.elsevier.com/retrieve/pii/S1769721221000604

5. Pejaver V, Urresti J, Lugo-Martinez J, Pagel KA, Lin GN, Nam H-J, et al. Inferring the molecular and phenotypic impact of amino acid variants with MutPred2. Nat Commun [Internet]. 2020 Dec 20;11(1):5918. Available from: http://mutpred.mutdb.org/

6. Shihab HA, Gough J, Cooper DN, Stenson PD, Barker GLA, Edwards KJ, et al. Predicting the Functional, Molecular, and Phenotypic Consequences of Amino Acid Substitutions using Hidden Markov Models. Hum Mutat [Internet]. 2013 Jan;34(1):57–65. Available from: http://doi.wiley.com/10.1002/humu.22225

7. Carter H, Douville C, Stenson PD, Cooper DN, Karchin R. Identifying Mendelian disease genes with the Variant Effect Scoring Tool. BMC Genomics [Internet]. 2013;14(Suppl 3):S3. Available from: http://www.biomedcentral.com/1471-2164/14/S3/S3

8. Douville C, Masica DL, Stenson PD, Cooper DN, Gygax DM, Kim R, et al. Assessing the Pathogenicity of Insertion and Deletion Variants with the Variant Effect Scoring Tool (VEST‐Indel). Hum Mutat [Internet]. 2016 Jan 26;37(1):28–35. Available from: https://onlinelibrary.wiley.com/doi/10.1002/humu.22911

9. Sim N-L, Kumar P, Hu J, Henikoff S, Schneider G, Ng PC. SIFT web server: predicting effects of amino acid substitutions on proteins. Nucleic Acids Res [Internet]. 2012 Jul 1;40(W1):W452–7. Available from: https://academic.oup.com/nar/article-lookup/doi/10.1093/nar/gks539

10. Adzhubei IA, Schmidt S, Peshkin L, Ramensky VE, Gerasimova A, Bork P, et al. A method and server for predicting damaging missense mutations. Nat Methods [Internet]. 2010 Apr;7(4):248–9. Available from: http://www.nature.com/articles/nmeth0410-248

11. Schwarz JM, Cooper DN, Schuelke M, Seelow D. MutationTaster2: mutation prediction for the deep-sequencing age. Nat Methods [Internet]. 2014 Apr 28;11(4):361–2. Available from: http://dx.doi.org/10.1038/nmeth.2890

12. Choi Y, Chan AP. PROVEAN web server: a tool to predict the functional effect of amino acid substitutions and indels. Bioinformatics [Internet]. 2015 Aug 15;31(16):2745–7. Available from: https://academic.oup.com/bioinformatics/article-lookup/doi/10.1093/bioinformatics/btv195

13. Reva B, Antipin Y, Sander C. Predicting the functional impact of protein mutations: application to cancer genomics. Nucleic Acids Res [Internet]. 2011 Sep;39(17):e118–e118. Available from: https://academic.oup.com/nar/article-lookup/doi/10.1093/nar/gkr407

14. Chun S, Fay JC. Identification of deleterious mutations within three human genomes. Genome Res [Internet]. 2009 Sep 1;19(9):1553–61. Available from: http://genome.cshlp.org/cgi/doi/10.1101/gr.092619.109

15. Davydov E V., Goode DL, Sirota M, Cooper GM, Sidow A, Batzoglou S. Identifying a High Fraction of the Human Genome to be under Selective Constraint Using GERP++. Wasserman WW, editor. PLoS Comput Biol [Internet]. 2010 Dec 2;6(12):e1001025. Available from: https://dx.plos.org/10.1371/journal.pcbi.1001025

16. Garber M, Guttman M, Clamp M, Zody MC, Friedman N, Xie X. Identifying novel constrained elements by exploiting biased substitution patterns. Bioinformatics [Internet]. 2009 Jun 15;25(12):i54–62. Available from: https://academic.oup.com/bioinformatics/article-lookup/doi/10.1093/bioinformatics/btp190

17. Ramani R, Krumholz K, Huang Y-F, Siepel A. PhastWeb: a web interface for evolutionary conservation scoring of multiple sequence alignments using phastCons and phyloP. Schwartz R, editor. Bioinformatics [Internet]. 2019 Jul 1;35(13):2320–2. Available from: https://academic.oup.com/bioinformatics/article/35/13/2320/5210871

18. Ioannidis NM, Rothstein JH, Pejaver V, Middha S, McDonnell SK, Baheti S, et al. REVEL: An Ensemble Method for Predicting the Pathogenicity of Rare Missense Variants. Am J Hum Genet [Internet]. 2016 Oct;99(4):877–85. Available from: http://dx.doi.org/10.1016/j.ajhg.2016.08.016

19. McLaren W, Gil L, Hunt SE, Riat HS, Ritchie GRS, Thormann A, et al. The Ensembl Variant Effect Predictor. Genome Biol [Internet]. 2016 Dec 6;17(1):122. Available from: http://dx.doi.org/10.1186/s13059-016-0974-4

20. Liu X, Jian X, Boerwinkle E. dbNSFP: A lightweight database of human nonsynonymous SNPs and their functional predictions. Hum Mutat [Internet]. 2011 Aug 26;32(8):894–9. Available from: https://onlinelibrary.wiley.com/doi/10.1002/humu.21517

21. Liu X, Li C, Mou C, Dong Y, Tu Y. dbNSFP v4: a comprehensive database of transcript-specific functional predictions and annotations for human nonsynonymous and splice-site SNVs. Genome Med [Internet]. 2020 Dec 2;12(1):103. Available from: https://genomemedicine.biomedcentral.com/articles/10.1186/s13073-020-00803-9

22. Culp M, Johnson K, Michailidis G. ada : An R Package for Stochastic Boosting. J Stat Softw [Internet]. 2006;17(2):1–27. Available from: http://www.jstatsoft.org/v17/i02/

23. Liaw A, Wiener M. Classification and Regression by randomForest. R news [Internet]. 2002;2(December):18–22. Available from: http://cran.r-project.org/doc/Rnews/

24. Yeo G, Burge CB. Maximum Entropy Modeling of Short Sequence Motifs with Applications to RNA Splicing Signals. J Comput Biol [Internet]. 2004 Mar;11(2–3):377–94. Available from: http://www.liebertpub.com/doi/10.1089/1066527041410418

25. Jian X, Boerwinkle E, Liu X. In silico prediction of splice-altering single nucleotide variants in the human genome. Nucleic Acids Res [Internet]. 2014 Dec 16;42(22):13534–44. Available from: http://academic.oup.com/nar/article/42/22/13534/2411339/In-silico-prediction-of-splicealtering-single

26. Brunak S, Engelbrecht J, Knudsen S. Prediction of human mRNA donor and acceptor sites from the DNA sequence. J Mol Biol [Internet]. 1991 Jul;220(1):49–65. Available from: https://linkinghub.elsevier.com/retrieve/pii/002228369190380O

27. Hebsgaard SM, Korning PG, Tolstrup N, Engelbrecht J, Rouzé P, Brunak S. Splice site prediction in Arabidopsis thaliana pre-mRNA by combining local and global sequence information. Nucleic Acids Res [Internet]. 1996 Sep 1;24(17):3439–52. Available from: https://academic.oup.com/nar/article-lookup/doi/10.1093/nar/24.17.3439

28. Krogh A. Two methods for improving performance of an HMM and their application for gene finding. Proceedings Int Conf Intell Syst Mol Biol [Internet]. 1997;5:179–86. Available from: http://www.ncbi.nlm.nih.gov/pubmed/9322033

29. Reese MG, Eeckman FH, Kulp D, Haussler D. Improved splice site detection in Genie. J Comput Biol [Internet]. 1997 Jan;4(3):311–23. Available from: http://www.liebertpub.com/doi/10.1089/cmb.1997.4.311

30. Desmet F-O, Hamroun D, Lalande M, Collod-Béroud G, Claustres M, Béroud C. Human Splicing Finder: an online bioinformatics tool to predict splicing signals. Nucleic Acids Res [Internet]. 2009 May;37(9):e67–e67. Available from: https://academic.oup.com/nar/article-lookup/doi/10.1093/nar/gkp215
