# Supplementary material for: Identification of Variants Responsible for Monogenic Forms of Diabetes in Brazil
Source: Front Endocrinol (Lausanne). 2022 May 3;13:827325. doi: 10.3389/fendo.2022.827325 (PMC9110842; doi:10.3389/fendo.2022.827325)
Supplement: Supplementary file 3 [file DataSheet_2.docx]

**Supplementary Tables**

**Supplemental Table S1.** Sequence of primers and PCR conditions

| **Gene** | **Primers** | **Sequences 5' 3'** | **Ta °C** | **Amplicon bp** | **Protocol** |
| --- | --- | --- | --- | --- | --- |
| ***GCK*** | E1F | GAAGGACACTAAGCCCCACAG | 60 | 505 | 1 |
|  | E1R | GGCACCCCTGGCAAGACC |  |  |  |
|  | E2F | GGGTCAGAAGACAGAAGGAGGC | 65 | 415 | 1 |
|  | E2R | CTGTCTCGGGCTGGCTGTG |  |  |  |
|  | E3F | CCTTAGTCCCTTGTGCCTTCC | 65 | 388 | 1 |
|  | E3R | CCGCTCTCCCCACCCCTG |  |  |  |
|  | E4F | CAGCAGAGCATTCAGCAGTATC | 60 | 690 | 1 |
|  | E4R | GGGGCTACATTTGAAGGCAGAG |  |  |  |
|  | E5-6F | CTCCAGTATATGTTAGCAGC | 60 | 504 | 1 |
|  | E5-6R | GATACCCCAAGACCACCCAGG |  |  |  |
|  | E7F | CACTGAAGCAACCCAGGTCT | 60 | 596 | 1 |
|  | E7R | GATCACCTGTCGGAAGGAAA |  |  |  |
|  | E8F | GAGGGAAAGACGTGAACCAG | 62 | 438 | 1 |
|  | E8R | AGGCCCTAGTTTCCCATCC |  |  |  |
|  | E9-10F | CTGTCGGAGCGACACTCA | 62 | 700 | 1 |
|  | E9-10R | ATGGAGCCTGGGTGCTGT |  |  |  |
| ***HNF1A*** | E1F | GTGGGTGCAAGGAGTTTGGT | 60 | 490 | 1 |
|  | E1R | GGCCCCTCTAGGCTCTCCT |  |  |  |
|  | E2F | GGGTTGACAAGGTTCCAGCA | 60 | 431 | 1 |
|  | E2R | TGCAGGTTGAATCCCACTGAC |  |  |  |
|  | E3F | AGGTCAGGGGAATGGACG | 60 | 353 | 1 |
|  | E3R | CTGGACAGCCTTTTACAGGACC |  |  |  |
|  | E4F | ACAGGGTTCCTCTGAGCCTG | 60 | 370 | 1 |
|  | E4R | TGACTGCTGTCACTGGGACA |  |  |  |
|  | E5F | AAGTGCTGAGGGCTGTGGA | 65 | 293 | 1 |
|  | E5R | CTGCTCCAGAATCTCCCTGC |  |  |  |
|  | E6F | AGGGAGATTCTGGAGCAGTCC | 60 | 369 | 1 |
|  | E6R | TGAGTCCCAGTGGCTCTTCC |  |  |  |
|  | E7F | GCTGTTTCCTGACCACCCT | 60 | 470 | 1 |
|  | E7R | CTGCAGACACCCTCAATCAC |  |  |  |
|  | E8-9F | CTGGGACTAGGGCTGTCAGG | 60 | 454 | 1 |
|  | E8-9R | ACAGTAAGGGAGGGGGTGGA |  |  |  |
|  | E10F | CTGCTGTGATCCAGGA | 60 | 414 | 1 |
|  | E10R | CCTCAGAGCCCTCCCTTTCT |  |  |  |
| ***HNF4A*** | E1F | ATCTTCCCAGAGGACGGTTT | 60 | 319 | 2 |
|  | E1R | CCAAAGATCTGCTCCTGGAC |  |  |  |
|  | E2F | AGGTGATGGAGTGGGAACAG | 60 | 396 | 2 |
|  | E2R | TCTGGGACCTACCCACTCAG |  |  |  |
|  | E3F | CGGGATGAAGAGATGAGAGC | 60 | 346 | 2 |
|  | E3R | TCTCAGCCATTAGCCAGTCA |  |  |  |
|  | E4F | GCTCCCACTCCTCATCAGTC | 60 | 342 | 2 |
|  | E4R | TGTGAAACCGGACTCAGTGT |  |  |  |
|  | E5F | CTCCCTCCCTCCGTTTTTAC | 60 | 406 | 2 |
|  | E5R | CCACGGCTATATCCCAGGT |  |  |  |
|  | E6F | TTCTTTCCCCTTCCAGGTTT | 60 | 418 | 2 |
|  | E6R | CTGGAGCCCTCTAATGCAAG |  |  |  |
|  | E7F | CCACAGGCACCAGCTATCTT | 60 | 392 | 2 |
|  | E7R | AAATGAAAACGGCCTCTCCT |  |  |  |
|  | E8F | ACAAGTCAGGGGACATCTGG | 60 | 391 | 2 |
|  | E8R | ACTGTGTGAGGCCTGTCTCC |  |  |  |
|  | E9F | TATTGGATGGGCTGGTTGAT | 60 | 378 | 2 |
|  | E9R | ACCCTGGAACCCAGAAAACT |  |  |  |
|  | E10F | AAAGGCTGGAATTTTGAGCA | 60 | 324 | 2 |
|  | E10R | CCTTCATCCTTCCCATTCCT |  |  |  |
| ***HNF1B*** | E1F | TTCCTGGATTTGGGGTTTGC | 60 | 556 | 3 |
|  | E1R | GGGACTTCTCTGGTGGGAAA |  |  |  |
|  | E2F | GCAGTCACCTTCTCCTCTGT | 60 | 417 | 3 |
|  | E2R | ACTTCAGGTTGAGGCAGAGG |  |  |  |
|  | E3F | TCCGTTGTCTGTCTGTCTGT | 56 | 498 | 2 |
|  | E3R | TTGATATTGGGGTTCTGTGGAA |  |  |  |
|  | E4F | CCCCTTCATACTCCCAACCA | 56 | 481 | 2 |
|  | E4R | ATTCTGGCAATGAGAGAGCG |  |  |  |
|  | E5F | TGGACAGGGGAGGAGAAAG | 56 | 398 | 2 |
|  | E5R | CCTATGGGGCTACAATGGTTC |  |  |  |
|  | E6F | CACCATGCCCAGCCAATAAT | 56 | 392 | 2 |
|  | E6R | TCGTGGGTGAGTTTGAAGGA |  |  |  |
|  | E7F | TAATGCCCATCTCCAACCCA | 60 | 479 | 3 |
|  | E7R | AGAGAGGGAAAGTGGTTGGC |  |  |  |
|  | E8F | AGATGGGAGCTATGGTGTGG | 59 | 334 | 2 |
|  | E8R | AACAACAGGGAGCCTCAGAA |  |  |  |
|  | E9F | AGAACTGAGCAGACACGAGG | 59 | 332 | 2 |
|  | E9R | AGTGGATTGTCTGAGGTGCC |  |  |  |
| ***INS*** | E1F | CCTTCAGCCTGCCTCAGC | 60 | 398 | 4 |
|  | E1R | CACTTTTAGGACGTGACCAAGA |  |  |  |
|  | E2F | TGGAGATGGGTGGGAGTG | 60 | 372 | 3 |
|  | E2R | AAGACACACAGACGGCACAG |  |  |  |
| ***NEUROD1*** | E1aF | GGTTTAGGGAGTGGAAGCTGA | 60 | 650 | 5 |
|  | E1aR | GTTGGTGGTGGGTTGGGATA |  |  |  |
|  | E1bF | TACATCTGGGCTCTGTCGGA | 60 | 676 | 3 |
|  | E1bR | TGTAAGCACAGTGGGTTCGT |  |  |  |
| ***KLF11*** | E1F | AGGGGCGCGGTGTATTTTG | 60 | 389 | 2 |
|  | E1R | CCCCACCTCCCGCATTTAC |  |  |  |
|  | E2F | TCGGTGTTTGTTGCTATAGACT | 60 | 500 | 2 |
|  | E2R | CCAGGGAATCTTCTCACAAGT |  |  |  |
|  | E3aF | AAGGTATTGGGAGCATTGTGA | 67 | 650 | 1 |
|  | E3aR | TCCAGTCACAGGGATCATCT |  |  |  |
|  | E3bF | GGTGTCCTGTCAGCCCTG | 68 | 648 | 1 |
|  | E3bR | AAAGGCGGCTCAAGGTGTG |  |  |  |
|  | E4F | AGTGTGGGAGGAATAAATGCC | 67 | 495 | 1 |
|  | E4R | AAAATCCCATGAGTGATGTCCT |  |  |  |
| ***KCNJ11*** | E1aF | AGAGTCTGGTGGGGAGTTATCT | 60 | 589 | 2 |
|  | E1aR | GGGCACTCCTCAGTCACC |  |  |  |
|  | E1bF | TCTTCACCATGTCCTTCCTGTG | 60 | 586 | 5 |
|  | E1bR | TCGTAGAGTGGGCTGTTGG |  |  |  |
|  | E1cF | TGGCCCCGCTGATCATCTA | 60 | 483 | 2 |
|  | E1cR | GCCGGGCTACATACCACAT |  |  |  |
| ***PAX4*** | E1F | GGGTATGGGCAAGGAACAAA | 60 | 493 | 2 |
|  | E1R | CCCCTTTTCAACCTCCGAGA |  |  |  |
|  | E2F | TGGCCTGGTCCAGTAAGTCT | 60 | 509 | 2 |
|  | E2R | GCTTTCTCCTTCCCAATCCT |  |  |  |
|  | E3F | AGGATTGGGAAGGAGAAAGCA | 63 | 473 | 2 |
|  | E3R | GGGACCTGTGTTCTGTTCCA |  |  |  |
|  | E4F | GGTATTGAGCACCCTTTCCA | 60 | 415 | 2 |
|  | E4R | CACTCACACCTGCACCTCTC |  |  |  |
|  | E5F | GTTTGGGGTTGTAGCAGGTG | 60 | 418 | 2 |
|  | E5R | CCCTCCCTGCTCTAGCTTTT |  |  |  |
|  | E6F | TCTTCCCCAACCCAAACCTT | 60 | 301 | 3 |
|  | E6R | GATAGATGACTGAGCGGGCA |  |  |  |
|  | E7F | AAGGACCTGTCTTGGGGAAG | 60 | 312 | 2 |
|  | E7R | GCTCAGGCCAGAAATGGAAG |  |  |  |
|  | E8F | ATACTACTTGGGTGGCAGGC | 60 | 502 | 3 |
|  | E8R | GGGAGAGAGGCTGAGACATC |  |  |  |
|  | E9F | CAGGGTGGGAAACTGATGTC | 60 | 447 | 3 |
|  | E9R | GTGAGAAGTGGGTGGGTGTT |  |  |  |
| ***MT-TL1*** | F | CCTCCCTGTACGAAAGGACA | 60 | 412 | 2 |
|  | R | GCGGTGATGTAGAGGGTGAT |  |  |  |

Ta: annealing temperature; bp: base pairs; F: Forward; R: Reverse; Protocol used for each reaction described above;

PCR conditions:

- **Protocol 1** - 50 ng of Genomic DNA, 1 unit of AmpliTaq DNA Polymerase (Applied Biosystems, Foster City, CA, USA), 1X PCR Buffer I (contains 15 mM MgCl_2_) (Applied Biosystems, Foster City, CA, USA), 0.2 mM of each dNTP and 0.4 μM of each primer.
- **Protocol 2 -** 50 ng of Genomic DNA, 1.5 unit of Taq DNA Polymerase Brasil (Invitrogen, CA, USA), 1X PCR Buffer (Invitrogen, CA, USA), 0.2 mM of each dNTP, 2 mM of MgCl_2_ and 0.4 μM of each primer.
- **Protocol 3** – 50 ng of Genomic DNA, 2 unit of FastStart Taq DNA Polymerase (Roche Diagnostics, Mannheim, Germany), 1X PCR Reaction Buffer (Roche Diagnostics, Mannheim, Germany), 2 mM of MgCl_2_, 1X GC-RICH Solution (Roche Diagnostics, Mannheim, Germany), 0.2 mM of each dNTP, 2 mM of MgCl_2_ and 0.4 μM of each primer.
- **Protocol 4 -** 50 ng of Genomic DNA, 1 unit of Platinum Taq DNA Polymerase High Fidelity (Invitrogen, CA, USA), 1X High fidelity PCR Buffer (Invitrogen, CA, USA), 0.2 mM of each dNTP, 2 mM of MgSO_4_ and 0.4 μM of each primer.
- **Protocol 5 –** 50 ng of Genomic DNA, 1.25 unit of AmpliTaq Gold™ DNA Polymerase (Applied Biosystems, CA, USA), 1X PCR Gold Buffer (Applied Biosystems, CA, USA), 0.2 mM of each dNTP, 2 mM of MgCl_2_ (Applied Biosystems, CA, USA), and 0.4 μM of each primer.

Cycling conditions:

- **Protocol 1** - 95 °C for 10 min, followed by 40 cycles of 94 °C for 1 min, gene-specific annealing temperature (°C) for 1 min and 72 °C for 1 min; and a final extension of 72 °C for 10 min.
- **Protocol 2 -** 95 °C for 10 min, followed by 40 cycles of 95 °C for 1 min, gene-specific annealing temperature (°C) for 1 min and 72 °C for 1 min; and a final extension of 72 °C for 10 min.
- **Protocol 3 -** 95 °C for 4 min, followed by 40 cycles of 95 °C for 30 s, gene-specific annealing temperature (°C) for 30 s and 72 °C for 1 min; and a final extension of 72 °C for 10 min.
- **Protocol 4 -** 96 °C for 9 min, followed by 40 cycles of 96 °C for 1min, gene-specific annealing temperature (°C) for 1 min and 30 s and 72 °C for 1 min; and a final extension of 72 °C for 10 min.
- **Protocol 5 -** 95 °C for 10 min, followed by 40 cycles of 94 °C for 1min, gene-specific annealing temperature (°C) for 1 min and 72 °C for 1 min; and a final extension of 72 °C for 10 min.

**Supplemental Table S2**. Brazilian patient’s clinical characteristics with variants in genes associated to monogenic diabetes

| **Patient** | **Sex** | **At diagnose:** | | **At entry in this study:** | | | | | | **Gene with mutation** | **Segregation study?** | **Parental origin of the mutation** |
| --- | --- | --- | --- | --- | --- | --- | --- | --- | --- | --- | --- | --- |
|  |  | **Age (years)** | **BMI (kg/m²)** | **Age (years)** | **BMI (kg/m²)** | **FPG (mg/dl)** | **HbA1c %** | **Treatment** | **Clinical diagnostic** |  |  |  |
|  |  |  |  |  |  |  |  |  |  |  |  |  |
| 45 | F | 24 | 19.2 | 40 | 21.6 | 110 | 6.5 | Diet | GCK-MODY | *GCK* | No | - |
| 46 | F | 25 | n/i | 35 | 23 | n/i | n/i | Diet | GCK-MODY | *GCK* | Yes | Father |
| 48 | M | 3 | < P95 | 10 | n/i | 120 | 6.8 | Ins | T1DM | *GCK* | No | - |
| 50 | M | 0.75 | n/i | 16 | 21 | 137 | 7 | Ins | T1DM | *GCK* | Yes | Mother |
| 53 | F | 15 | n/i | 31 | 19.2 | 112 | 6 | Diet | MODY | *GCK* | Yes | Mother |
| 55 | M | 26 | 22.3 | 32 | 26.1 | 109 | 6.1 | Diet | n/i | *GCK* | Yes | Father |
| 58 | M | 8 | < P85 | 13 | 16.4 | 122 | n/i | Diet | T1DM | *GCK* | Yes | Mother |
| 59 | F | 12 | < P85 | 22 | 19.8 | 130 | 6.1 | OAD | n/i | *GCK* | No | - |
| 63 | M | 5 | n/i | 10 | 18.9 | 118 | 6.3 | Ins | n/i | *GCK* | Yes | Mother |
| 67 | M | 3 | n/i | 16 | 19.8 | 120 | n/i | Diet | GCK-MODY | *GCK* | Yes | Mother |
| 68 | F | 21 | 19.3 | 35 | 18.1 | n/i | 6.2 | OAD | MODY | *GCK* | No | - |
| 75 | F | 8 | < P85 | 13 | 20.8 | 126 | 6.4 | Diet | n/i | *GCK* | Yes | Father |
| 79 | M | 5 | < P85 | 17 | 19.6 | 139 | 7.2 | OAD | T1DM | *GCK* | No | - |
| 44 | M | 21 | 23.8 | 34 | n/i | n/i | 7 | Ins | n/i | *HNF1A* | Yes | - |
| 52 | F | 15 | n/i | 49 | 25.8 | n/i | 8.3 | OAD + Ins | T2DM | *HNF1A* | Yes | - |
| 56 | F | 35 | 28.2 | 35 | 25.3 | 133 | 6.9 | Ins | n/i | *HNF1A* | No | - |
| 70 | F | 25 | 28.8 | 27 | 28 | 154 | 7 | OAD | T2DM | *HNF1A* | No | - |
| 23 | F | 19 | 20 | 49 | 20.7 | 294 | 9.3 | OAD | n/i | *HNF4A* | Yes |  |
| 65 | F | 14 | 23.6 | 19 | 21.9 | > 500 | 12.1 | Ins | T1DM | *HNF1B* | No |  |
| 26 | M | 28 | 25.9 | 30 | 20 | 116 | 5.6 | OAD | T1DM | *MT-TL1* | No | - |

F: Female; M: Male; AAD: Age at diagnosis; P: Percentile; BMI: Body Mass Index; OAD: Oral antidiabetic agents; FPG: Fasting Plasma Glucose; HbA1c: Glycated hemoglobin; T1DM: Type 1 diabetes *mellitus*; T2DM: Type 2 diabetes *mellitus*; n/i: Not informed.

**Supplemental Table S3.** *In silico* algorithms prediction for the variants identified in the Brazilian patients described by our group

| **Variants** | | **Prediction algorithms** | | | | | | | | | | | | | |
| --- | --- | --- | --- | --- | --- | --- | --- | --- | --- | --- | --- | --- | --- | --- | --- |
|  |  | **Pathogenicity** | | | | | | | | | **Conservation** | | | |  |
| **Gene** | **Variants**  **(protein level)** | **SIFT ^a^** | **PROVEAN ^b^** | **VEST ^c^** | **MutPred ^d^** | **FATHMM ^e^** | **PolyPhen-2 (HumVar) ^f^** | **Mutation Assessor ^g^** | **Mutation Taster ^h^** | **LRT ^i^** | **GERP++ ^j^** | **SiPhy ^k^** | **phyloP_mammalian ^l^** | **phast Cons ^m^** | **Revel ^n^** |
| ***GCK***  ***HNF1A*** | p.(Arg36Trp) | D | D | LP | P | D | PbD | M | DC | Dt | Nc | C | C | C | P |
|  | p.(Arg43His) | D | D | LP | P | D | PbD | M | DC | Dt | C | C | C | C | P |
|  | p.(Gly44Ser) | D | D | LP | P | D | PbD | H | DC | Dt | C | C | C | C | P |
|  | p.(Met115Val) | D | N | LP | P | D | PbD | M | DC | Dt | C | C | C | C | P |
|  | p.(Ala188Thr) | D | D | LP | P | D | PbD | M | DC | Dt | C | C | C | Nc | P |
|  | p.(Thr209Arg) | D | D | LP | P | D | PbD | H | DC | Dt | C | C | C | C | P |
|  | p.(Glu221Lys) | T | D | LP | P | D | PbD | L | DC | Dt | C | C | C | C | P |
|  | p.(Pro359Leu) | T | N | LP | P | D | PsD | M | DC | Dt | C | C | C | C | P |
|  | p.(Phe423Tyr) | D | N | LP | P | D | PbD | L | DC | Dt | C | C | C | C | P |
|  | p.(Asp365GlufsTer95) | D | - | LP | - | - | - | - | - | - | - | - | - | - | - |
|  | p.(Lys39del) | D | D | LP | - | - | - | - | - | - | - | - | - | - | - |
|  | p.(Arg271Trp) | D | D | LP | P | D | PbD | M | DC | Dt | C | Nc | C | C | P |
|  | p.(Tyr163Ter) | - | - | LP | - | - | - | - | A | Dt | C | Nc | C | C | - |
|  | p.(Val380CysfsTer39) | D | - | LP | - | - | - | - | - | - | - | - | - | - | - |
| ***HNF4A*** | p.(Arg163Ter) | - | - | LP | - | - | - | - | A | N | C | C | C | C | - |
| ***HNF1B*** | p.(Arg276Ter) | - | - | LP | - | - | - | - | A | Dt | C | C | C | C | - |

D: Damaging; T: Tolerated; P: Pathogenic; DC: Disease causing; A: Disease causing automatic; Dt: Deleterious; N: Neutral; L: Low impact; M: Medium impact; H: High impact; PbD: Probably damaging; PsD: Possibly damaging; LP: Likely pathogenic; C: Conserved; Nc: Nonconserved; -: Not analyzed.

^a^ SIFT score < 0.05 is predicted as damaging.

^b^ PROVEAN score ≤ -2.5 is predicted as damaging and > - 2.5 as neutral.

^c^ VEST score ranges from 0 to 1, the larger the score the more likely the mutation may cause functional change.

^d^ Mutation Assessor score ≤ 0.8 is predicted to have neutral impact , between > 0.8 and 1.9 low impact , between > 1.9 to ≤ 3.5 medium impact , and > 3.5 high impact .

^e^ MutPred score range from 0 to 1, the larger the score the more likely the damaging effect.

^f^ FATHMM score ≤ -1.5 is predicted as damaging and > -1.5 is predicted as tolerated.

^g^ Polyphen-2_HumVAR score between 0.909 and 1 is predicted as probably damaging, between 0.447 and 0.908 is predicted as possibly damaging, between 0 and 0.446 is predicted as benign.

^h^ MutationTaster prediction: A: disease causing automatic, D: disease causing, N: polymorphism or P: polymorphism automatic. The score cutoff between "D" and "N" is 0.5.

^I^ LRT score ranges from 0 to 1 and which is not solely determined by the score. It is predicted as deleterious, neutral or unknown.

^J^ GERP++ scores range from -12.3 to 6.17 the larger the score, the more conserved the site. The score cutoff between nonconserved and conserved is ≥ 2,0.

^k^ SiPhy score based on 29 mammals genomes. The larger the score, the more conserved the site. Scores range from 0 to 37.9718 in dbNSFP. The score cutoff between nonconserved and conserved is ≥ 12.17 (56).

^l^ PhyloP score based on 30 mammalian genomes (including human). The larger the score, the more conserved the site. Scores range from -20 to 1.312 in dbNSFP. The score cutoff between nonconserved and conserved is ≥ 0.

^m^ PhastCons score based on the multiple alignments of 30 mammalian genomes (including human). The larger the score, the more conserved the site. The score cutoff between nonconserved and conserved is ≥ 0.5.

^n^ Revel score is based on 13 individual scores for predicting the pathogenicity of missense variants. Scores range from 0 to 1. The larger the score the more likely the SNP has damaging effect.

**Supplemental Table S4.** Description of the variants identified in the Brazilian patients described by our group in public database

| **Variant** | **dbSNP** | **HGMD** | **gnomAD**  **(allele frequency)** | **ClinVar** | **ACMG/**  **AMP** |
| --- | --- | --- | --- | --- | --- |
| *GCK* p.(Arg36Trp) | rs762263694 | Yes | Yes (0.00001414) | P, LP | P |
| *GCK* p.(Lys39del) | - | - | - | US | LP |
| *GCK* p.(Arg43His) | rs764232985 | Yes | Yes (0.0000039771) | LP | P |
| *GCK* p.(Gly44Ser) | rs267601516 | Yes | - | P | P |
| *GCK* p.(Met115Val) | rs771677681 | - | Yes (0.00001193) | - | LP |
| *GCK* p.(Ala188Thr) | rs751279776 | Yes | Yes (0.000003982) | P | LP |
| *GCK* p.(Thr209Arg) | - | - | - | - | P |
| *GCK* p.(Glu221Lys) | rs193922317 | Yes | - | P, LP | P |
| *GCK* p.(Pro359Leu) | - | Yes | - | - | LP |
| *GCK* p.(Asp365GlufsTer95) | - | - | - | - | P |
| *GCK* p.(Phe423Tyr) | rs193922273 | Yes | - | LP | LP |
| *HNF1A* p.(Tyr163Ter) | - | - | - | - | P |
| *HNF1A* p.(Arg271Trp) | rs886039386 | Yes | - | P | LP |
| *HNF1A* p.(Val380CysfsTer39) | - | - | - | - | P |
| *HNF4A* p.(Arg163Ter) | rs137853335 | Yes | - | P | P |
| *HNF1B* p.(Arg276Ter) | rs121918672 | Yes | - | P | P |
| *MT-TL1* m.3243A>G | rs199474657 | - | - | P | US |

P: Pathogenic; LP: Likely pathogenic; US: Uncertain significance; -: Not available.
